# Intravenous immunoglobulin therapy in COVID-19-related encephalopathy

**DOI:** 10.1007/s00415-020-10248-0

**Published:** 2020-10-08

**Authors:** Lorenzo Muccioli, Umberto Pensato, Giorgia Bernabè, Lorenzo Ferri, Maria Tappatà, Lilia Volpi, Ilaria Cani, Olivia J. Henry, Francesca Ceccaroni, Sabina Cevoli, Gloria Stofella, Elena Pasini, Giacomo Fornaro, Caterina Tonon, Simone Vidale, Rocco Liguori, Paolo Tinuper, Roberto Michelucci, Pietro Cortelli, Francesca Bisulli

**Affiliations:** 1 Department of Biomedical and Neuromotor Sciences, University of Bologna, Bellaria Hospital, Via Altura 3 40139, Bologna, Italy; 2 Infermi Hospital, AUSL Romagna, Rimini, Italy; 3 IRCCS Istituto delle Scienze Neurologiche di Bologna, Bologna, Italy; 4 Karolinska Institutet, Stockholm, Sweden; 5 Department of Medical and Surgical Sciences, University of Bologna, Bologna, Italy

## Abstract

**Objective:**

To report on efficacy and safety of intravenous immunoglobulin (IVIg) therapy in a case series of patients with COVID-19-related encephalopathy.

**Methods:**

We retrospectively collected data on all patients with COVID-19 hospitalized at two Italian hospitals who developed encephalopathy during disease course and were treated with IVIg.

**Results:**

Five patients (two females, mean age 66.8 years) developed encephalopathy after a mean of 12.6 days, since the onset of respiratory/constitutional symptoms related to COVID-19. Four patients suffered severe respiratory distress, three of which required invasive mechanical ventilation. Neurological manifestations included impaired consciousness, agitation, delirium, pyramidal and extrapyramidal signs. EEG demonstrated diffuse slowing in all patients. Brain MRI showed non-specific findings. CSF analysis revealed normal cell count and protein levels. In all subjects, RT-PCR for SARS-CoV-2 in CSF tested negative. IVIg at 0.4 g/kg/die was commenced 29.8 days (mean, range: 19–55 days) after encephalopathy onset, leading to complete electroclinical recovery in all patients, with an initial improvement of neuropsychiatric symptoms observed in 3.4 days (mean, range: 1–10 days). No adverse events related to IVIg were observed.

**Conclusions:**

Our preliminary findings suggest that IVIg may represent a safe and effective treatment for COVID-19-associated encephalopathy. Clinical efficacy may be driven by the anti-inflammatory action of IVIg, associated with its anti-cytokine qualities.

**Electronic supplementary material:**

The online version of this article (10.1007/s00415-020-10248-0) contains supplementary material, which is available to authorized users.

## Introduction

Severe acute respiratory syndrome-coronavirus-2 (SARS-CoV-2) is the causative agent of coronavirus disease-2019 (COVID-19). While many individuals with SARS-CoV-2 infection are asymptomatic or develop only mild respiratory and constitutional symptoms, a subgroup of patients present with complications, including acute respiratory distress syndrome, disseminated intravascular coagulation and multiorgan dysfunction syndrome [1, 2].

Cytokine release syndrome (CRS) is a systemic hyperinflammatory condition presenting secondary to monocyte, macrophage and dendritic cell activation in severe COVID-19 infection and has been implicated in disease pathophysiology [3].

Neuropsychiatric manifestations are increasingly being reported in association with COVID-19, including encephalopathy [4]. The pathophysiology underlying this presentation remains unclear; however, a role of cytokine-mediated neuroinflammation has been suggested [5−10]. COVID-19-associated encephalopathy has been described responsive to high-dose steroids and plasmapheresis, consistent with an immune-mediated pathogenesis [5, 11, 12].

Intravenous immunoglobulin (IVIg) therapy has shown efficacy in treating systemic COVID-[13, 19] yet its role in the management of associated CNS manifestations remains to be determined.

We report five patients with COVID-19-related encephalopathy successfully treated with IVIg.

## Methods

We retrospectively collected data on all patients with COVID-19 hospitalized at Bellaria Hospital, Bologna, and Infermi Hospital, Rimini, Italy, from March 13, 2020, to May 27, 2020, who developed encephalopathy during disease course and were treated with IVIg. COVID-19 diagnosis was made on the basis of at least one positive SARS-CoV-2 real-time reverse-transcriptase-polymerase-chain-reaction (RT-PCR) assay of nasopharyngeal swab specimens and consistent clinical and/or radiological findings. IVIg therapy was prescribed by the treating neurologist based on the patient’s clinical profile and suspected immune-mediated/inflammatory encephalopathy, in accordance with institutional and international guidelines.

## Results

**Fig. 1:**
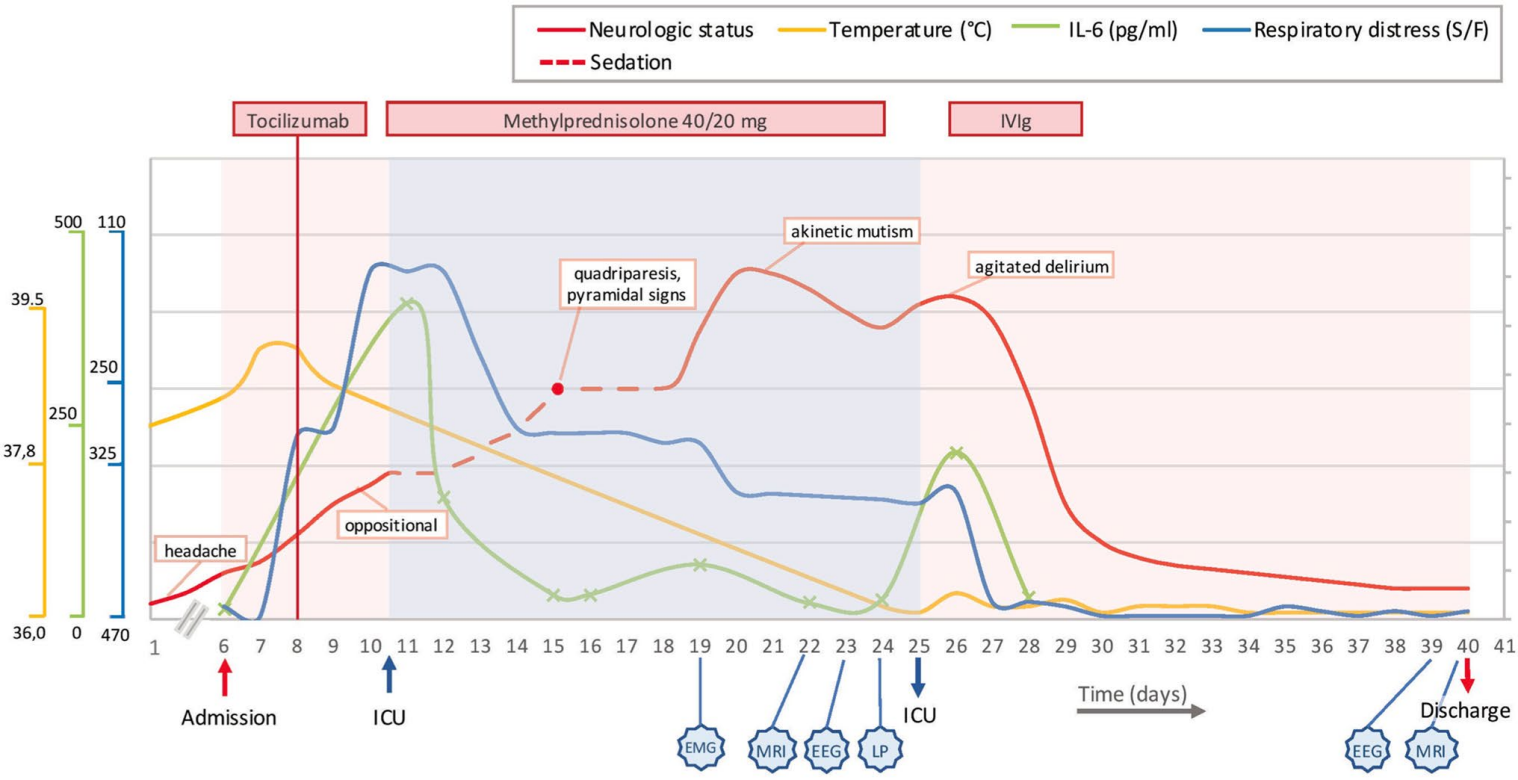
Disease course in patient 1. Neurological manifestations, respiratory distress, temperature, IL-6 levels, timing of immunomodulatory treatments and diagnostic investigations during disease course. Neurological status severity was evaluated by treating neurologists. On the 15th day after disease onset, neurological evaluation was performed during temporary weaning of sedatives (red dot). Temperature and SpO2/FiO2 ratio were measured at least daily during hospitalization. Crosses on the IL-6 line represent the actual measurements. *EEG* electroencephalography, *EMG* electromyography, *ICU* intensive care unit, *IVIg* intravenous immunoglobulin treatment, *LP* lumbar puncture, *MRI* magnetic resonance imaging, *S/F* SpO2/FiO2 ratio

Five patients (two females) with a mean age of 66.8 years (range: 54–75 years) were included in this retrospective study. Demographics, comorbidities, disease course, timing of IVIg and other immunotherapies are summarized in Table 1. Clinical, neuroradiological, EEG and CSF findings are summarized in Table 2. The illustrative case of the first patient is shown in Fig. 1.

**Table 1:** Demographics, comorbidities and disease course

Pt	Age (y), Sex	Comorbidities	COVID-19 onset (day)^a,b^	Worst P/F (day)^a^	IVIg treatment (0.4 g/kg/d) (start, end)^a^	Clinical Response (initial, complete)^a^	Other immunotherapies (start, end)^a^	Last f.up (day)^a^
1	54, F	None	− 5	130 (+ 3)	+ 18, + 21	+ 19, + 21	Tocilizumab 400 mg (+ 0) Low-dose steroids (+ 3, + 17)	+ 90
2	75, M	Type 2 DM, hypertension, ischemic heart disease, previous stroke	0	114 (+ 12)	+ 26, + 30	+ 28, + 34	Tocilizumab 400 mg (+ 33) Low-dose steroids (+ 21, + 36)	+ 115
3	69, F	Bipolar disorder, MCI, iatrogenic parkinsonism, type 2 DM	− 15	345 (+ 14)	+ 28, + 32	+ 30, + 38	MP 1 g/die (+ 13, + 17)	+ 86
4	69, M	Hypertensive cardiopathy	− 23	81 (− 14)	+ 22, + 27	+ 24, + 28		+ 70
5	67, M	Type 2 DM, hypertension	− 20	79 (− 8)	+ 55, + 60	+ 65, + 75	Tocilizumab 400 mg (− 8)	+ 105

*DM* diabetes mellitus, *MCI* mild cognitive impairment, *MP* methylprednysolone

^a^We referred to encephalopathy onset as day “0”, and to all events occurred previously or subsequently as minus or plus “day”, respectively

^b^Onset of constitutional or respiratory symptoms such as fever, cough, dyspnea

**Table 2:** Neurological clinical and investigative findings

Pt	Neurological manifestations	EEG (D)	MRI (D)	CSF (D)
1	Irritability, quadriparesis with pyramidal signs, akinetic mutism, agitated delirium, frontal release reflexes	Diffuse slowing at 6–7 Hz (+ 15)	Fronto-parietal white matter hyperintensity (+ 16)	Cells: 0/μL Proteins: 26 mg/dL Qalb: 4 (+ 17)
2	Confusion, disorientation, global memory deficits	Diffuse slowing at 4–5 Hz (+ 21)	Previous right fronto-parietal stroke (+ 22)	Cells: 1 WBC/μL Proteins: 60 mg/dL Qalb: 17.8 (+ 25)
3	Apraxia, mixed delirium, pyramidal signs, frontal release reflexes, extrapyramidal signs (rigidity and bradykinesia)^a^	Diffuse slowing at 6–7 Hz, frontal sharp waves (+ 13)	Parietal white matter hyperintensity, cerebral atrophy (+ 13)	Cells: 1 WBC/μL Proteins: 32 mg/dL Qalb: 3.8 (+ 9)
4	Decreased level of consciousness, agitation, tonic muscle spasms	Diffuse slowing at 5–6 Hz, FIRDA (+ 0)	Cerebral small vessel disease (chronic) (+ 2)	Cells: 6 WBC/μL Proteins 35 mg/dL (+ 1)
5	Decreased level of consciousness, agitation, hemiparesis with pyramidal signs, extrapyramidal signs (rigidity and tremor), frontal release reflexes	Diffuse slowing at 5–6 Hz (+ 2)	Cerebral small vessel disease (chronic) (+ 45)	Cells: 1 WBC/μL Proteins: 29 mg/dL (+ 2)

*D* number of days after encephalopathy onset, *FIRDA* frontal intermittent rhythmic delta activity, *Qalb* CSF/serum albumin quotient, *WBC* white blood cell

^a^Extrapyramidal signs were already present before COVID-19 due to drug-induced parkinsonism

Three patients (1, 4, 5) were placed on invasive mechanical ventilation due to respiratory distress, while patient 2 was treated with non-invasive mechanical ventilation. Patient 3 had prior hospitalization in another facility for mild respiratory distress, where she was treated with low-flow oxygen therapy. She was subsequently re-admitted to our facility due to the subacute onset of neuropsychiatric symptoms. All patients developed encephalopathy, with a mean onset of 12.6 days (range 0–23 days) after the presentation of respiratory/constitutional symptoms related to SARS-CoV-2 infection. In patients 4 and 5, encephalopathy presented following sedation weaning as persisting impaired consciousness, whereas CNS symptoms developed in the other patients independently. Neurological manifestations were heterogeneous, including confusion, agitation, delirium, akinetic mutism, apraxia, pyramidal, extrapyramidal and frontal release signs. EEG demonstrated diffuse slowing in all patients, with frontal predominance in two cases. Brain MRI showed non-specific diffuse white-matter hyperintensities in two patients, while the others displayed chronic cerebrovascular findings. CSF analysis revealed ≤ 6 WBC and normal protein levels in each patient. In all subjects, RT-PCR for SARS-CoV-2 and for common neurotropic viruses in CSF, as well as a panel of CSF and serologic antibodies against neuronal intracellular and cell surface antigens, tested negative. Blood tests revealed elevated inflammatory markers, including IL-6 (Supplementary Appendix). Patient 3 developed hypernatremia (up to 164 mEq/L) and prerenal acute kidney injury during hospitalization, but their correction did not result in mental status improvement. With this exception, common causes of encephalopathy such as renal or hepatic failure, electrolyte imbalances, and hypoxemia were excluded in all cases. IVIg at 0.4 g/kg/die was commenced 29.8 days (mean, range 19–55 days) after encephalopathy onset, leading to complete electroclinical recovery in all patients, with an initial improvement of neuropsychiatric symptoms observed in 3.4 days (mean, range 1–10 days). No further neurological symptoms were observed at the last follow-up visit, which was performed after a mean of 54.8 days (range 30–81) since clinical recovery. In patient 1, a marked reduction of serum IL-6 levels was evident on the fourth IVIg infusion day (from 218 to 28 pg/mL), coinciding with clinical response. No adverse events related to IVIg were observed.

## Discussion

Encephalopathy is emerging as a recurrent complication of COVID-19, yet the best approach to management and treatment remains unknown. We described five patients with COVID-19-related encephalopathy, all of whom recovered following IVIg.

IVIg is an efficient anti-inflammatory and immunomodulatory treatment for a growing number of neurological disorders; however, its mode of action is complex and not yet fully understood [15]. The pathophysiology underlying the diseases which respond to IVIg is highly heterogeneous, thus it is likely that IVIg acts on various disease-specific pathways.

In patients with COVID-19 and severe pulmonary involvement, treatment with IVIg led to a significant clinical improvement and concomitant reduction of serum inflammatory markers, observed as early as the first infusion day [13]. Correspondingly, our patients showed a prompt and dramatic improvement of neurological manifestations following IVIg. Similar results have been achieved in six other patients with COVID-19-related encephalopathy treated with high-dose corticosteroids [5, 11]. In our third case, steroid pulse therapy was ineffective, where subsequent IVIg was concomitant with clinical recovery. In support of our preliminary findings, IVIg led to improved neuropsychiatric symptoms in four COVID-19 patients with mixed central and peripheral neurological manifestations and to clinico-radiological recovery in a further patient with frontal status epilepticus and encephalopathy [16, 17].

Clinical course and investigative findings observed in the present case series, including negative RT-PCR for SARS-CoV-2 in CSF, absence of significant elevation of CSF cells and protein levels, non-specific MRI findings, and the dramatic response to immunotherapy, suggest an inflammatory/immune-mediated pathogenesis rather than CNS viral invasion. An autoantibody-mediated mechanism is unlikely to explain CNS involvement, based on the brief temporal interval between CNS and infection-related symptom onset, negative testing for anti-neuronal antibodies, and the prompt and sustained response to IVIg [15]. Cytokine-mediated neuroinflammation has been implicated in the underlying pathogenic mechanism of COVID-19-associated encephalopathy, and may have contributed to disease course in our patients [5–10]. Except the third case, all patients suffered acute respiratory distress secondary to CRS induced by SARS-CoV-2. Interestingly, case 3 is the only patient with previous cognitive impairment, a condition that possibly made her more susceptible to develop encephalopathy despite mild systemic inflammation, as in other reported geriatric patients [10]. In patient 1 and 4, we observed a delay between the rise of inflammatory markers in serum and the onset/peak of neurological manifestations, possibly due to a delayed rise of CSF cytokines with respect to serum levels. Unfortunately, we were not able to measure CSF cytokines.

IVIg has anti-cytokine qualities, which appear important for its anti-inflammatory action. This may be related to the presence of anti-cytokine autoantibodies found in natural human immunoglobulin, including anti-TNF-α, anti-IL-1 and anti IL-8 [18]. Additionally, the natural IgG fraction inhibits the production of proinflammatory cytokines in a dose-dependent manner via the Fc portion [18]. Accordingly, IVIg therapy has been shown to significantly reduce circulating proinflammatory cytokines within 3 days [19], as observed in patient 1.

Cytokines may drive neuroinflammation even without severe CRS, possibly as a consequence of local CNS production [5, 6, 20]. The IVIg anti-cytokine effects documented peripherally may independently and directly act on the CNS. IVIg can cross an intact blood–brain-barrier and is bioavailable in sufficient concentrations to interact with the therapeutic targets, with the maximum concentration reached in the CNS 24 h after administration in a murine model [1, 2].

None of our patients experienced adverse events related to IVIg. Adverse reactions to IVIg therapy are usually minor and occur in less than 10% of patients [15]. However, as both COVID-19 and IVIg may predispose to thromboembolic events such as stroke and pulmonary embolism [2, 15], prophylactic anticoagulation should be considered. In patients with evidence of coagulopathy, an alternate immunotherapy such as high-dose corticosteroids should be preferred. However, since the likelihood of thromboembolic disease in COVID-19 might be secondary to CRS, the global thromboembolic risk may be partially compensated by the anti-inflammatory action of IVIg [1, 16].

## Conclusions

IVIg was a safe and effective treatment for COVID-19-related encephalopathy in our case series. Clinical efficacy may be driven by the anti-inflammatory action of IVIg, associated with its anti-cytokine qualities. Our preliminary observation needs to be confirmed with larger clinical studies. However, considering the emerging evidence supporting an inflammatory-mediated pathogenesis in a subgroup of patients with COVID-19-related encephalopathy, we believe that immunotherapy with IVIg or other agents might be considered to hasten clinical recovery and prevent potential long-term neurological sequelae.

## Electronic supplementary material

Below is the link to the electronic supplementary material.

Supplementary material 1 (DOCX 22 kb)
