# Supplementary material for: Intravenous immunoglobulin therapy in COVID-19-related encephalopathy
Source: J Neurol. 2020 Oct 8;268(8):2671–5. doi: 10.1007/s00415-020-10248-0 (PMC7543032; doi:10.1007/s00415-020-10248-0)
Supplement: Supplementary file 1 — Supplementary material 1 (DOCX 22 kb) [file 415_2020_10248_MOESM1_ESM.docx]

**Supplementary Table 1 – Blood tests in patient 1**

|  | *Reference values* | *+3* | *+18* | *+21* |
| --- | --- | --- | --- | --- |
| **Hematology**  White-cell count (10^9^/L)  Neutrophil count (10^9^/L)  Lymphocyte count (10^9^/L)  Hemoglobin (g/dL)  Platelet count (10^9^/L) | 3.6-10.5  1.5-7.7  1.1-4  12.0-15.6  160-370 | 8.18  7.42  0.52  10.9  275 | 7.20  4.64  1.94  10.1  322 | 5.75  3.33  1.96  9.7  227 |
| **Coagulation**  INR  aPTT  Fibrinogen (mg/dL) | <1.2  0.82-1.25  150-400 | 1.12  0.88  524 | 1.17  0.79  481 | NA  NA  NA |
| **Biochemistry**  Sodium (mmol/L)  Potassium (mmol/L)  Glucose (mg/dL)  Urea (mg/dL)  Creatinine (mg/dL)  eGFR (mL/min)  Total protein (g/dL)  Albumin (g/L)  Total bilirubin (mg/dL)  AST (U/L)  ALT (U/L)  CPK (U/L) | 136-145  3.5-5.3  60-110  17-43  0.5-1.2  NA  6.6-8.3  35-50  <1.2  <35  <35  <145 | 141  3.8  105  33  0.61  103  5.2  25.9  0.3  32  19  38 | 148  4.0  89  NA  0.43  115  6.2  NA  0.57  23  50  44 | 140  3.2  77  NA  045  114  7.1  0.45  30  56  107 |
| **Serum inflammatory proteins**  C-reactive protein (mg/dL)  IL-6 (pg/mL)  Procalcitonin (ng/mL)  Ferritin (ng/mL)  LDH (U/L) | <0.5  <5.9  <0.5  11-306  <248 | 3.9  412  <0.1  126  613 | 0.55  218  <0.1  68  243 | 0.13  28.2  <0.1  62  222 |

**Supplementary Table 2 – Blood tests in patient 2**

|  | *Reference values* | *+12* | +23 (+21) | +33 |
| --- | --- | --- | --- | --- |
| **Hematology**  White-cell count (10^9^/L)  Neutrophil count (10^9^/L)  Lymphocyte count (10^9^/L)  Hemoglobin (g/dL)  Platelet count (10^9^/L) | 3.6-10.5  1.5-7.7  1.1-4  12.0-15.6  160-370 | 7.55  6.09  0.86  16.1  196 | 7.96  6.07  1.09  15.9  164 | 8.17  5.34  2.24  15.5  196 |
| **Coagulation**  INR  aPTT  Fibrinogen (mg/dL) | <1.2  0.82-1.25  150-400 | NA  NA  NA | 1.08  1.08  360 | NA  NA  NA |
| **Biochemistry**  Sodium (mmol/L)  Potassium (mmol/L)  Glucose (mg/dL)  Urea (mg/dL)  Creatinine (mg/dL)  eGFR (mL/min)  Total protein (g/dL)  Albumin (g/L)  Total bilirubin (mg/dL)  AST (U/L)  ALT (U/L)  CPK (U/L) | 136-145  3.5-5.3  60-110  17-43  0.5-1.2  NA  6.6-8.3  35-50  <1.2  <35  <35  <145 | 141  4.2  144  NA  0.86  85  6.9  NA  0.79  113  148  56 | 143  3.9  168  45  1  73  (6.8)  NA  (1.29)  25  51  27 | 135  4.5  168  69  1.04  70  NA  33.6  1.18  27  46  NA |
| **Serum inflammatory proteins**  C-reactive protein (mg/dL)  IL-6 (pg/mL)  Procalcitonin (ng/mL)  Ferritin (ng/mL)  LDH (U/L) | <0.5  <5.9  <0.5  11-306  <248 | 16.9  48.6  <0.1  1019  394 | 1.18  97.2  0.1  575  NA | 0.49  NA  NA  NA  NA |

**Supplementary Table 3 – Blood tests in patient 3**

|  | *Reference values* | *+14* | +27 | +33 |
| --- | --- | --- | --- | --- |
| **Hematology**  White-cell count (10^9^/L)  Neutrophil count (10^9^/L)  Lymphocyte count (10^9^/L)  Hemoglobin (g/dL)  Platelet count (10^9^/L) | 3.6-10.5  1.5-7.7  1.1-4  12.0-15.6  160-370 | 7.82  6.92  0.76  10.7  184 | 5.04  3.86  0.92  9.4  168 | 3.66  2.53  0.94  9.9  137 |
| **Coagulation**  INR  aPTT  Fibrinogen (mg/dL) | <1.2  0.82-1.25  150-400 | 1.03  0.82  290 | 1.02  0.86  481 | NA  NA  NA |
| **Biochemistry**  Sodium (mmol/L)  Potassium (mmol/L)  Glucose (mg/dL)  Urea (mg/dL)  Creatinine (mg/dL)  eGFR (mL/min)  Total protein (g/dL)  Albumin (g/L)  Total bilirubin (mg/dL)  AST (U/L)  ALT (U/L)  CPK (U/L) | 136-145  3.5-5.3  60-110  17-43  0.5-1.2  NA  6.6-8.3  35-50  <1.2  <35  <35  <145 | 162  3.6  350  89  1.82  29.4  5.5  25.9  0.35  25  38  299 | 150  3.5  117  NA  0.72  85  5.1  NA  0.47  18  35  35 | 144  3.3  77  NA  0.76  80  NA  NA  NA  NA  NA  NA |
| **Serum inflammatory proteins**  C-reactive protein (mg/dL)  IL-6 (pg/mL)  Procalcitonin (ng/mL)  Ferritin (ng/mL)  LDH (U/L) | <0.5  <5.9  <0.5  11-306  <248 | 0.07  24.8  0.2  80  NA | 2.15  26.4  <0.1  68  285 | 1.85  17.5  NA  NA  NA |

**Supplementary Table 4 – Blood tests in patient 4**

|  | *Reference values* | -14 | +22 | +27 |
| --- | --- | --- | --- | --- |
| **Hematology**  White-cell count (10^9^/L)  Neutrophil count (10^9^/L)  Lymphocyte count (10^9^/L)  Hemoglobin (g/dL)  Platelet count (10^9^/L) | 3.6-10.5  1.5-7.7  1.1-4  12.0-15.6  160-370 | 6.39  5.40  0.51  11.4  166 | 5.46  3.40  1.49  9.3  158 | 8.53  5.98  1.93  9.4  186 |
| **Coagulation**  INR  aPTT  Fibrinogen (mg/dL) | <1.2  0.82-1.25  150-400 | 0.99  0.91  315 | NA  NA  NA | 1.12  1.05  460 |
| **Biochemistry**  Sodium (mmol/L)  Potassium (mmol/L)  Glucose (mg/dL)  Urea (mg/dL)  Creatinine (mg/dL)  eGFR (mL/min)  Total protein (g/dL)  Albumin (g/L)  Total bilirubin (mg/dL)  AST (U/L)  ALT (U/L)  CPK (U/L) | 136-145  3.5-5.3  60-110  17-43  0.5-1.2  NA  6.6-8.3  35-50  <1.2  <35  <35  <145 | 134  4.0  150  42  0.45  117  NA  NA  0.3  34  38  43 | 137  3.5  NA  34  0.22  >120  NA  NA  0.3  21  31  15 | 138  3.7  NA  19  0.21  >120  NA  NA  NA  25  33  14 |
| **Serum inflammatory proteins**  C-reactive protein (mg/dL)  IL-6 (pg/mL)  Procalcitonin (ng/mL)  Ferritin (ng/mL)  LDH (U/L) | <0.5  <5.9  <0.5  11-306  <248 | 8.4  >1000  0.05  NA  251 | 23.9  24.0  0.04  NA  193 | 24.6  NA  0.04  NA  170 |

**Supplementary Table 5 – Blood tests in patient 5**

|  | *Reference values* | -8 | +55 | +65 |
| --- | --- | --- | --- | --- |
| **Hematology**  White-cell count (10^9^/L)  Neutrophil count (10^9^/L)  Lymphocyte count (10^9^/L)  Hemoglobin (g/dL)  Platelet count (10^9^/L) | 3.6-10.5  1.5-7.7  1.1-4  12.0-15.6  160-370 | 2.58  2.15  0.32  10.8  296 | 4.84  2.18  2.01  8.9  197 | 3.86  1.09  2.08  8.7  170 |
| **Coagulation**  INR  aPTT  Fibrinogen (mg/dL) | <1.2  0.82-1.25  150-400 | 1.13  0.83  483 | 1.12  1.02  411 | 1.10  1.06  371 |
| **Biochemistry**  Sodium (mmol/L)  Potassium (mmol/L)  Glucose (mg/dL)  Urea (mg/dL)  Creatinine (mg/dL)  eGFR (mL/min)  Total protein (g/dL)  Albumin (g/L)  Total bilirubin (mg/dL)  AST (U/L)  ALT (U/L)  CPK (U/L) | 136-145  3.5-5.3  60-110  17-43  0.5-1.2  NA  6.6-8.3  35-50  <1.2  <35  <35  <145 | 140  4.6  107  36  0.57  106  NA  28  0.25  38  31  31 | 142  4.8  102  12  0.24  >120  NA  NA  0.33  18  14  13 | 131  3.5  130  19  0.28  >120  NA  NA  0.31  25  21  20 |
| **Serum inflammatory proteins**  C-reactive protein (mg/dL)  IL-6 (pg/mL)  Procalcitonin (ng/mL)  Ferritin (ng/mL)  LDH (U/L) | <0.5  <5.9  <0.5  11-306  <248 | 49.9  6.7  0.04  274  183 | 11.9  NA  0.04  NA  164 | 11.9  NA  0.02  NA  NA |
